# Removal of Motion Artifacts in Photoplethysmograph Sensors during Intensive Exercise for Accurate Heart Rate Calculation Based on Frequency Estimation and Notch Filtering

**DOI:** 10.3390/s19153312

**Published:** 2019-07-28

**Authors:** Min Wang, Zhe Li, Qirui Zhang, Guoxing Wang

**Affiliations:** 1 Department of Micro/Nano Electronics, Shanghai Jiao Tong University, Shanghai 200240, China; 2 Academy of Information Technology and Electrical Engineering, Shanghai Jiao Tong University, Shanghai 200240, China; 3 Department of Electrical Engineering and Computer Science, University of Michigan, Ann Arbor, MI 48109, USA

**Keywords:** adaptive noise cancellation (ANC), heart rate estimation, motion artifact removal, notch filtering, photoplethysmography (PPG)

## Abstract

With photoplethysmograph (PPG) sensors showing increasing potential in wearable health monitoring, the challenging problem of motion artifact (MA) removal during intensive exercise has become a popular research topic. In this study, a novel method that combines heart rate frequency (HRF) estimation and notch filtering is proposed. The proposed method applies a cascaded adaptive noise cancellation (ANC) based on the least mean squares (LMS)-Newton algorithm for preliminary motion artifacts reduction, and further adopts special heart rate frequency tracking and correction schemes for accurate HRF estimation. Finally, notch filters are employed to restore the PPG signal with estimated HRF based on its quasi-periodicity. On an open source data set that features intensive running exercise, the proposed method achieves a competitive mean average absolute error (AAE) result of 0.92 bpm for HR estimation. The practical experiments are carried out with the PPG evaluation platform developed by ourselves. Under three different intensive motion patterns, a 0.89 bpm average AAE result is achieved with the average correlation coefficient between recovered PPG signal and reference PPG signal reaching 0.86. The experimental results demonstrate the effectiveness of the proposed method for accurate HR estimation and robust MA removal in PPG during intensive exercise.

## 1. Introduction

Photoplethysmography (PPG) has proven effective in monitoring cardiovascular-related physiological signs, especially heart rate (HR), oxygen saturation (SpO_2_) and blood pressure (BP) [1]. Due to the advantages of low cost and convenience, PPG sensors are widely applied in wearable healthcare. Though having a great potential for wearable healthcare, the accuracy of PPG sensors during motion such as exercise by the user is still unsatisfactory due to motion artifacts (MA) [2]. The MA is typically caused by the change of blood flow velocity induced by the motion [3] or the relative movement between PPG sensors and human skin [4]. The wide frequency range of MA with time-varying nature makes it difficult to use traditional filtering techniques for the removal of motion artifacts [5]. Thus, to improve the reliability and accuracy of health monitors based on PPG sensors, the removal of MA continues to be a technical challenge that needs to be tackled.

Early studies attempted to reconstruct PPG signal from MA-corrupted signals with traditional signal processing methods. Independent component analysis (ICA) is a common method [6], which can separate motion artifacts and clean PPG signal from multi-channel corrupted PPG signals. However, if the statistical independence between motion artifacts and clean PPG signals is not well satisfied, the method could hardly be effective [7]. Researchers have also adopted wavelet transform for PPG MA removal [8]. Nevertheless, this method needs users to empirically set the threshold values for wavelet de-noising, which limits its usage. The empirical mode decomposition (EMD) was also proposed for removing motion artifacts [9], but the performance of EMD may be affected by intermittency signals and noise, known as the mode-mixing problem. Later, more researchers tried to combine different techniques to achieve better results. In [10], researchers proposed a method that combines Fourier series reconstruction and frequency-domain independent component analysis (FD-ICA) to reduce PPG motion artifacts in a step-by-step manner. Another method proposed in [11] combines multiple signal processing techniques and adaptive noise cancellation (ANC), which uses fast Fourier transform (FFT), singular value decomposition (SVD) and ICA to extract the noise reference from corrupted PPG signal. However, these methods are still not effective for restoring the PPG signal from a heavily corrupted signal.

Recently, some application-oriented studies tried to skip exact PPG signal recovery and directly estimated the physiological signs from preliminary de-noised PPG signal during intensive exercise, especially in the application of HR monitoring [12]. In [13], researchers combine sparse signal reconstruction (SSR) and a special HR tracking scheme to accurately estimate HR from de-noised PPG signal. The method proposed in [14] applies an empirical and complex tracking scheme that incorporates the ensemble empirical mode decomposition (EEMD) algorithm to estimate HR. Another method proposed in [15] combines the phase vocoder technique, a HR tracking and a smoothing stage for HR estimation from de-noised PPG signal. There are also many other methods which follow a similar routine as the ones mentioned above. Although such methods may be able to estimate HR from MA-corrupted PPG signal accurately, it could not be applicable to other physiological signs estimation, such as BP monitoring, because these signs need to extract more comprehensive information from PPG signal, which has stricter requirements for signal quality.

However, because the useful part of PPG signal is mainly constructed by spectral components around its fundamental frequency and harmonic frequencies [16], the accurate estimation of HR can be a key for restoring PPG signal due to the high correlation between heart rate frequency (HRF) and fundamental frequency of PPG. In this paper, a robust method is proposed for removing motion artifacts in PPG signals during intensive exercise. The method combines accurate HRF estimation with notch filtering. It first reduces the motion artifacts through a cascaded ANC stage that filters the corrupted PPG signal using three-axis acceleration signals. Then a simple heart rate frequency tracking (HRFT) scheme and a specially-designed heart rate frequency correction (HRFC) stage are applied for HRF estimation. After that, two tunable notch filters are constructed using the HRF and its second harmonic frequency to restore the PPG signal. Finally, the estimated HR value and the restored PPG signal are obtained by the proposed method.

## 2. Proposed Method

**Figure 1:**
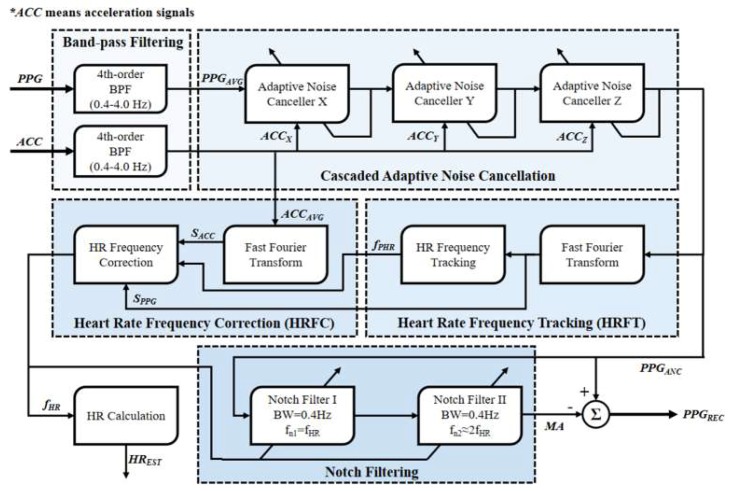
Overall signal flow of the proposed method.

Shown in Figure 1 is the overall signal flow of the proposed method. The method requires PPG signal(s) and three-axis acceleration signal(s) as its inputs. The 4th order 0.4 Hz–4.0 Hz Butterworth band-pass filters (BPF) are firstly employed to remove the out-of-band noise of the input PPG and acceleration signals. Then a cascaded ANC stage is adopted to adaptively cancel the motion artifacts in the PPG signals using respectively x, y and z-axis acceleration signals as reference noise. After that, a simple HRFT scheme is applied to give a preliminary estimation for the HRF from the spectrum of the ANC-de-noised PPG signal. Then a novel HRFC stage is used to correct possibly wrong HRF estimation and give the final estimated HRF value. The HRF and its second harmonics frequency are used as notch frequencies to construct two notch filters. The notch filters remove the PPG components from the ANC-de-noised PPG signal and extract the motion artifacts. And the MA-removed PPG signal is finally obtained through subtracting the motion artifacts from the ANC-de-noised PPG signal.

A widely-utilized open source data set provided for the 2015 IEEE Signal Processing Cup [12] is used for preliminary evaluation of different stages in the proposed method. The data set contains signals collected from 12 subjects (11 males and one female). For each subject, two channels of PPG signals and three-axis acceleration signals are recorded from one wrist, with one channel electrocardiograph (ECG) signal recorded simultaneously for about 5 min with a sampling rate of 125 Hz. As the ECG signal is barely influenced by MA, it is used for calculating the reference HR. During the 5 min recording, the subjects exercise on a treadmill in the following pattern: 1–2 km/h walking for 30 s, 6–8 km/h running for 60 s, 12–15 km/h running for 60 s, 6–8 km/h running for 60 s, 12–15 km/h running for 60 s and 1–2 km/h walking for 30 s. For the first 30 s, subjects are in a relatively static initialization stage. Average Absolute Error (AAE) between reference HR and estimated HR from the proposed method [12] is used for evaluating performance of the proposed method, which denoted as *HR_AAE_*. The *HR_AAE_* for each subject is calculated using Equation (1). *HR_EST_(i)* and *HR_TRUE_(i)* are respectively the *i*-th HR estimation results and reference HR value. *N* is the number of total estimations for a subject.

(1) $$HR_{AAE}=\frac{1}{N}\overset{N}{\underset{i=1}{\sum }}\left|HR_{EST}\left(i\right)-HR_{TRUE}\left(i\right)\right|.$$

### 2.1. Cascaded Adaptive Noise Cancellation

**Figure 2:**
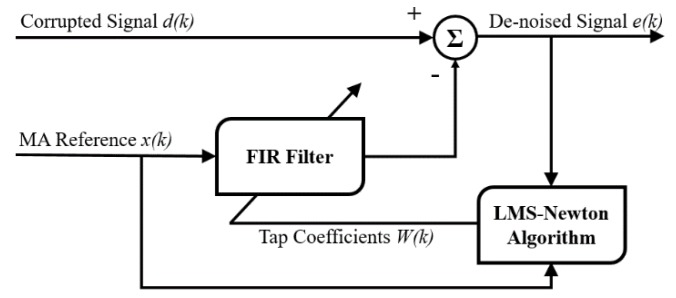
Signal flow of adaptive noise cancellation.

Adaptive noise cancellation is an effective technique to de-noise a noise-corrupted signal given a reference signal that is highly correlated with the noise [17]. Figure 2 shows the signal flow of typical ANC. In the proposed method, the least mean squares-Newton (LMS-Newton) algorithm [18] is applied for adaptive filtering, which features a faster convergence speed than conventional LMS-based algorithms. The LMS-Newton algorithm is given in Equations (2) and (3). The parameter *x(k)* is the reference signal of MA, which is the acceleration signal. *W(k)* is the weight vector of the finite impulse response (FIR) filter, and *e(k)* is the output for de-noised PPG signal. The parameter α and *μ* in Equation (3) determine the speed of convergence.

(2) $$R^{-1}\left(k\right)=\frac{1}{1-\alpha }\left[R^{-1}\left(k-1\right)-\frac{R^{-1}\left(k-1\right)X\left(k\right)X^{T}\left(k\right)R^{-1}\left(k-1\right)}{\frac{1-\alpha }{\alpha }+X^{T}\left(k\right)R^{-1}\left(k-1\right)X\left(k\right)}\right]$$

(3) $$W\left(k+1\right)=W\left(k\right)+2\mu e\left(k\right)R^{-1}\left(k\right)X\left(k\right)$$

Because MA can be decomposed into motions in different directions, a cascaded topology of ANC is used here to reduce the motion artifacts better, as is illustrated in Figure 1. The cascaded adaptive noise cancellers use respectively x, y and z-axis acceleration signals as their MA reference inputs. Band-pass filtered PPG and acceleration signals are streamed into this step at a specified time interval *ltv*. In order to avoid an unmatched scale between the PPG signal and the acceleration signal, Z-score standardization is adopted to normalize the inputs of each adaptive noise canceller. Especially, if there are multiple PPG signals at the input end of the cascaded ANC, the PPG signals will be averaged into a single PPG signal before the first adaptive noise canceller.

Shown in Table 1 is the experimental results of *HR_AAE_* before and after applying the cascaded ANC stage of the proposed method. When only BPF is applied to the MA-corrupted signals, the estimation errors for HR values are very large, reaching an average *HR_AAE_* of 11.47 bpm over the 12 subjects, which indicates that the MA frequencies are strongly deviated from the HRFs and the spectral magnitudes of MA are larger. After applying the cascade ANC stage for MA reduction, the HR estimation errors significantly decreased.

### 2.2. Heart Rate Frequency Tracking

After the cascaded ANC stage, the de-noised PPG signal *S_PPG_* is used for a preliminary estimation of current HRF. FFT is first applied to acquire the spectrum of *S_PPG_*, where only the magnitude information is considered. A relatively large number of points for FFT *L_FFT_* is used to increase frequency resolution. Then a simple HRFT scheme from [15] is used for preliminary HRF estimation.

**Figure 3:**
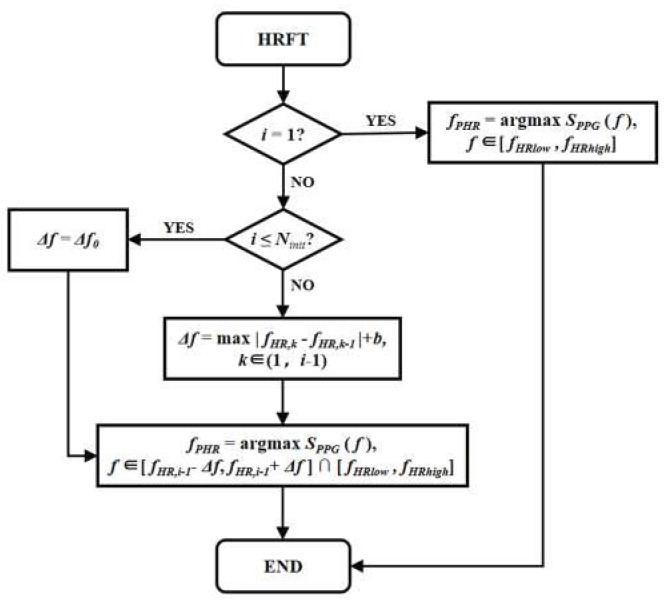
Flowchart of heart rate frequency tracking

Flowchart of the HRFT scheme is shown in Figure 3. The variable *i* corresponds to the *i*-th PPG signal sequence being processed by the proposed algorithm since it starts. *f_HRlow_* and *f_HRhigh_* are the low and high boundaries of typical human HRF. The partameter *f_PHR_* denotes the value of preliminary HRF estimation result and *f_HR_* is the final estimated HRF value. For the first signal sequence, *f_PHR_* is directly selected to be the frequency that corresponds to the largest spectral magnitude of *S_PPG_* within the human HRF range. Starting from the second signal sequence, *f_PHR_* is tracked from a range of ±*Δf* around *f_HR_* of the previous sequence. When *i* is smaller or equivalent to *N_init_*, *Δf* is set to be an initial constant *Δf0*. *N_init_* corresponds to the initialization stage of the proposed algorithm that typically takes 30 s to 1 min, where the users are required to stay motionless. If the initialization stage has finished, *Δf* is updated according to be the sum of the largest absolute difference of previous consecutive HRF estimation results and a bias *b*. Then, *f_PHR_* is selected to be corresponding to the largest spectral magnitude within a range of ±*Δf* around previous *f_HR_* and also within human HRF range. The calculated *HR_AAE_* results after applying the HRFT stage have been improved, as shown in Table 2.

### 2.3. Heart Rate Frequency Correction

**Figure 4:**
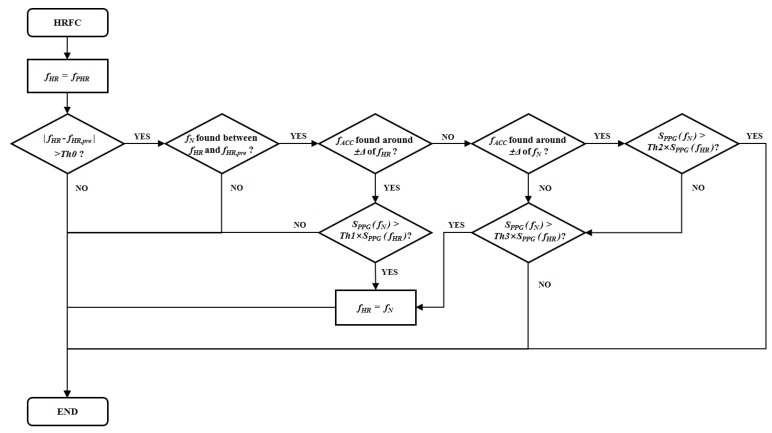
Flowchart of the heart rate frequency correction.

After the preliminary HRF estimation, a heuristic HRF correction scheme is further designed to correct possibly wrong *f_PHR_* results and give the final HRF estimation result *f_HR_*. The flow of this scheme is illustrated in Figure 4. The value of *f_HR_* is initialized as *f_PHR_*. For a relatively small time interval, the change in human HRF is not expected to be very large. So if the difference between current *f_HR_* and HRF estimation result of the previous PPG signal sequence *f_HR,pre_* becomes larger than a threshold *Th0*, it is highly possible that either *f_HR_* or *f_HR;pre_* is a wrong estimation result. Under that circumstance, the HRFC scheme tries to judge whether *f_HR_* is estimated to be wrong and will correct it if so (As the proposed method targets real-time online application, *f_HR,pre_* should not be corrected).

To correct a possibly wrong *f_HR_* result, the HRFC stage first tries to find *f_N_*, which corresponds to the largest current PPG spectral peak between *f_HR_* and *f_PHR_*. If *f_N_* is found, it is highly possible that *f_N_* is the correct HRF estimation result for *f_HR_*. However, it need to further check its fidelity to make the final decision. It first needs to check whether *f_ACC_* is within a small range of ±Δ around *f_HR_*, where *f_ACC_* is the frequency of the largest spectral magnitude of *S_ACC_*, and *S_ACC_* is the magnitude spectrum of acceleration signal calculated from the average of the three-axis acceleration sequences. If that is true, *f_HR_* is highly possible to be a wrong result which actually corresponds to motion artifacts, and HRFC further checks whether the spectral magnitude of *f_N_* is large enough when compared to the one of *f_HR_* so that *f_N_* can be the correct estimation result of *f_HR_*.

If *f_ACC_* is not found to be around *f_HR_*, HRFC cannot decide whether *f_HR_* is a wrong result, and it further checks whether *f_N_* can be a faked HRF peak which is actually caused by motion artifacts. If *f_ACC_* is found to be around *f_N_* and the spectral magnitude of *f_N_* is not large enough, HRFC keeps the value of *f_HR_* unchanged. If HRFC is not able to make a decision after all those checks, it simply compares the spectral magnitude of *f_N_* and *f_HR_* and set *f_HR_* to be *f_N_* if the magnitude of *f_N_* is large enough. For the spectral magnitude comparisons between *f_N_* and *f_HR_*, the range of thresholds *Th1*, *Th2* and *Th3* is (0, 1). After final estimation of HRF, the HR value (60 *f_HR_* bpm) for current PPG sequence is also calculated as an output of the proposed method.

As shown in Table 3, the final average *HR_AAE_* over the 12 subjects is 0.92 bpm for the complete HRF estimation algorithm, with an average standard deviation (SD) of 1.50 bpm (SDs are calculated within the estimation results of every subjects and then averaged over the 12 subjects).

### 2.4. Notch Filtering

**Figure 5:**
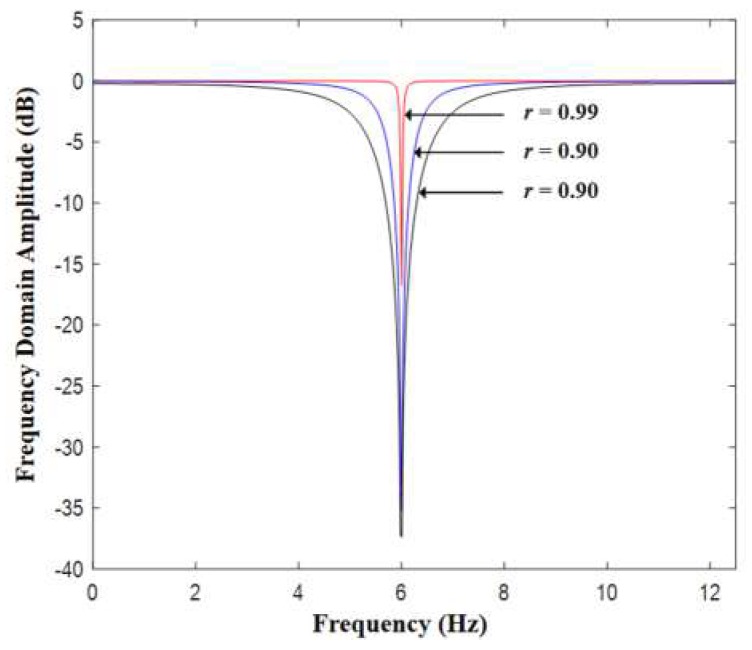
Frequency domain amplitude response of a notch filter.

Cascaded notch filters are used to restore the PPG signal based on the estimated HRF. As is discussed above, the PPG signal can be treated as a quasi-periodic signal with spectral components mainly distributed as peaks around its HRF and the harmonic frequencies of HRF. Thus notch filters or comb filters can be used to eliminate unwanted components and keep only the PPG-related ones. Shown in Equation (5) is the typical transfer function of a digital notch filter [19]. In Equation (4), *f_n_* is the notch frequency and *F_s_* is signal sampling rate. Shown in Figure 5 is the frequency domain amplitude response of the notch filter, where the parameter *r* controls its bandwidth. It can be observed from Figure 5 that notch filters actually suppress the signal around notch frequencies while keep the components of other frequencies close to their original amplitudes.

(4) $$\omega_{n}=\frac{2\pi f_{n}}{F_{s}}$$

(5) $$H\left(z\right)=\frac{r^{2}-\left(1+r^{2}\right)\cos\left(\omega_{n}\right)z^{-1}+z^{-2}}{1-\left(1+r^{2}\right)\cos\left(\omega_{n}\right)z^{-1}+r^{2}z^{-2}}.$$

For every processing iteration (every *Itv* s), two notch filters are constructed using respectively current HRF estimation result *f_HR_* and the second harmonic frequency of *f_HR_*, because the main features of time domain PPG signal in one period are its main pulse and dicrotic pulse. For the first notch filter, notch frequency is set to be *f_HR_* directly, while for the second one, its notch frequency is set to be the one that corresponds to the largest spectral peak around 2 *f_HR_* due to the quasi-periodicity of PPG signals. The output of the cascaded ANC stage is filtered using the two notch filters, where the PPG components are removed while the MA is kept as it is. Through finally subtracting the notch filtering output from the ANC-de-noised PPG signal, the restored PPG signal are achieved.

**Figure 6:**
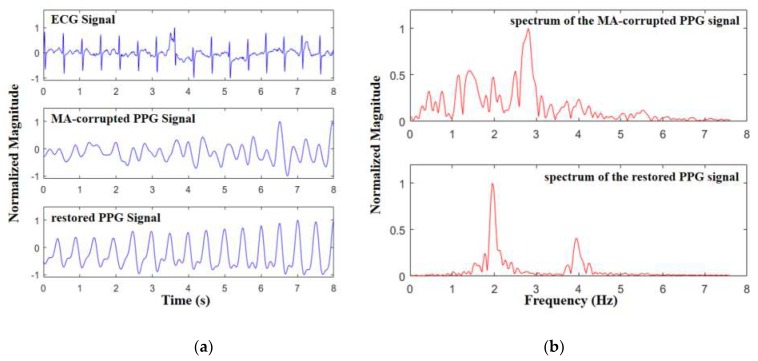
The performance of the proposed method to restore photoplethysmography (PPG) signal: (**a**) comparison of PPG signals before and after the proposed algorithm in time domain, with electrocardiograph (ECG) signal as reference; (**b**) comparison of PPG signals before and after the proposed algorithm in frequency domain.

Figure 6 shows the performance of the proposed method to restore the PPG signal from MA-corrupted signal. The PPG signal was seriously distorted during intensive exercise, and the amplitude of the PPG component in the frequency domain was not obvious. After the proposed method was applied, the waveform of the PPG signal was restored and only the fundamental and second harmonic frequency of the PPG signal were found in the frequency domain. Because there was no reference PPG signal in the open source data set, a preliminary judgment was made through ECG that the peak number and interval of the recovered PPG signal are consistent with that of ECG.

## 3. Experimental Results

### 3.1. Preliminary Evaluation of HR Estimation Based on Open Source Data Set

For comparison with other published works, the data set and rules from 2015 IEEE Signal Processing Cup were used. The parameters for the open source data set are set as the following: *L_p_* (prefix signal window length) = 990; δ (positive constant used to initialize ANC filter) = 400, *M* (ANC filter order) = 33, *α* = 2 × 10^−4^ and *μ* = 9 × 10^−5^; *L_FFT_* = 65536, Δ*f_0_* = 0.42 Hz (25 bpm), *N_init_* = 30 (60 s) and *b* = 0.08 Hz (5 bpm); *Th0* = 0.08 Hz (5 bpm), *Δ* = 0.07 Hz (4 bpm), *Th1* = 0.4, *Th2* = 0.9 and *Th3* = 0.85.

Shown in Table 4 is the *HR_AAE_* experimental results of the proposed method, with comparison to other published state-of-art methods from 2016 to 2018 that use the same data set for evaluation. And the proposed method achieves the lowest error, which shows the strength of the proposed method for accurate HR estimation during intensive exercise.

**Figure 7:**
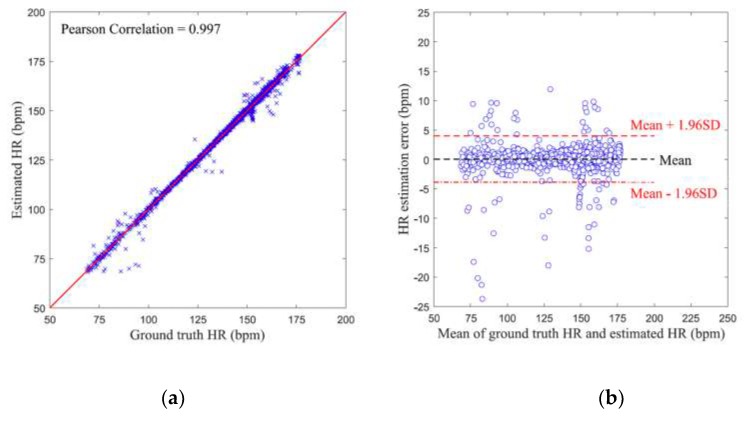
The evaluation of the difference between heart rate (HR) estimation results and the reference HR values: (**a**) Pearson correlation plot for *HR_AAE_* results; (**b**) Bland–Altman plot for *HR_AAE_* results.

Figure 7a shows the Pearson correlation plot for all the HR estimation results. The Pearson correlation coefficient reached 0.997, showing that the estimated HR values were highly correlated with the reference HR values. Figure 7b shows the Bland-Altman plot [23] of all the HR estimation results for the proposed method, which illustrates the relationship between HR estimation errors and the mean of estimated HR and reference HR. It can be shown that though the largest HR estimation error reaches around 25 bpm, 95% of the errors are within the range of [–4 bpm, 4 bpm], which indicated a high agreement between the estimated HR values and reference HR values.

### 3.2. Evaluation under Practical Experiment

Due to the lack of reference PPG signals in open source data set, we cannot evaluate the results of signal recovery well. To further verify the performance of the proposed method, a PPG evaluation platform is developed to set up the self-built data set including reference PPG signals, and then demonstrate the effectiveness of the proposed algorithm under practical environment.

#### 3.2.1. Implementation of Evaluation Hardware and Software

**Figure 8:**
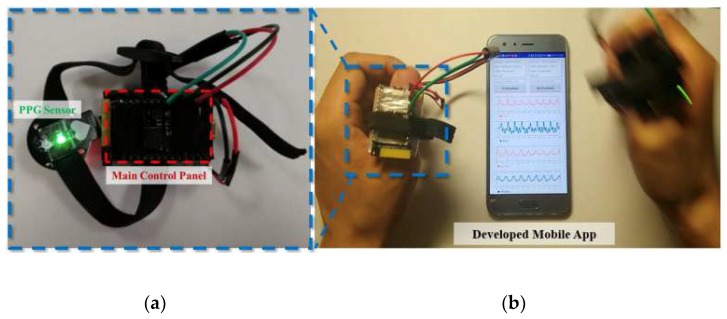
The developed PPG evaluation platform: (**a**) the signal acquisition finger-band system consisting of the PPG sensor and main control panel; (**b**) demonstration of the developed platform: the subject will wear the finger-band on both hands of them, with the left hand stationary and the right hand moving.

Figure 8 shows the developed PPG evaluation platform, which consists of two signal acquisition finger-bands and a mobile software application. The finger-bands can simultaneously record one channel PPG signal and three-axis acceleration signals with 25 Hz sampling rate, in which the PPG sensor (SEN0203, DFRobot, China) was used to obtain PPG signal with green LEDs and the main control panel (CurieNano, Intel, USA) integrates the functions of 32-bit MCU, 12-bit ADC, six-axis motion sensor and Bluetooth Low Energy (BLE). The Figure 8a shows the signal acquisition finger-band. The software application developed with JAVA was installed on the android smartphone equipped with Octa-core (4 × 2.4 GHz Cortex-A73 and 4 × 1.8 GHz Cortex-A53), which was used to receive data from the two finger-bands and run the proposed algorithm. The restored PPG signals and estimated HR values were displayed on the mobile phone. After testing, the average running time of the proposed algorithm in the developed platform is 83 ms (the average result of 60 runs of the algorithm), which shows a great real-time performance. The parameters of the algorithm realized in the software application are set as the following: *L_p_* =203; δ = 400, *M* = 8, *α*= 1.0 × 10^−4^ and *μ* = 4.5 × 10^−5^; *L_FFT_* = 4096, Δ*f0* = 0.42 Hz (25 bpm), *N_init_* = 30 (60 s) and *b* = 0.08 Hz (5 bpm); *Th0* = 0.08 Hz (5 bpm), *Δ* = 0.07 Hz (4 bpm), *Th1* = 0.4, *Th2* = 0.9 and *Th3* = 0.85.

#### 3.2.2. Experimental Process

During experiment, the subject will wear two finger-bands with each of them on one thumb, as shown in Figure 8b. The left hand and arm are kept motionless, where only one channel PPG signal is recorded as the reference PPG signal with no motion artifacts. The right hand and arm performed certain movements, where one channel MA-corrupted PPG signal and corresponding three-axis acceleration signals were recorded. As left and right hands and arms were symmetric, the pure PPG signals collected from them should be highly similar. Thus the PPG signal collected from motionless left hand can be used as a reference PPG signal, which can indicate the effectiveness of our proposed method.

All the raw data mentioned above and processed results of estimated HR values and restored PPG signals are displayed on the mobile phone in real time and saved as self-built data set. A total of 20 subsets of data from 13 male subjects aging from 20 to 23 are collected, with their duration time and motion patterns shown in Table 5. The first subset is a control group where the subject stays motionless for 120 s. The second motion pattern featured relatively regular large-joint movements, while the third pattern was mainly composed of random small-joint movements.

#### 3.2.3. Experiment Results

The correlation coefficient between MA-removed PPG and reference PPG signal [24] was adopted for evaluating the performance of the proposed method to restore the PPG signal. The correlation between two PPG signal sequences *S_1_* and *S_2_* can be calculated using Equation (6), where *L* is the length of the output PPG sequence. *μ_1_* and *μ_2_* are the averages of *S_1_* and *S_2_*. *σ_1_* and *σ_2_* are the standard deviations. The typical range of correlation coefficient was [–1, 1]. A higher value of the absolute correlation means a higher similarity in two signal sequences. In this study, we denote the correlation between MA-removed PPG and reference PPG as *CORR_aMAR_*. Denoted as *CORR_bMAR_*, the correlation coefficient between MA-corrupted PPG signal and reference PPG signal is also used for showing the improvement in PPG signal purity.

(6) $$Corr\left(S_{1},S_{2}\right)=\frac{\sum_{i=1}^{L}(S_{1}\left(i\right)-\mu_{1})\left(S_{2}\left(i\right)-\mu_{2}\right)}{L\sigma_{1}\sigma_{2}}$$

Table 6 shows the experimental results for the 20 data subsets of self-built data set. The reference PPG signal or MA-corrupted PPG signal are first filtered using the BPF in Figure 1 to avoid the influence of out-of-band noise to the experimental results. In the respect of PPG signal recovery, the result of control group (subset 1) shows the correlation between the PPG signals from left hand and right hand is quite good, which means the left-hand signal can be treated as a reference for the right-hand indeed. The average *CORR_bMAR_* over the 20 subsets was 0.57, which is significantly lower than unity, showing that right-hand PPG signals were strongly corrupted by motion artifacts and the waveforms are distorted. After applying the proposed MA-removal method, the average correlation is increased to 0.86, which is close to the value of control group, indicating that PPG signal waveforms have been recovered well. In respect of HR estimation, the reference HR values were directly calculated from the magnitude spectrums of reference PPG signals. With the complete proposed method, the overall average *HR_AAE_* is 0.89 bpm, which shows the robustness of the proposed method for accurate HR estimation during different types of intensive exercise.

**Figure 9:**
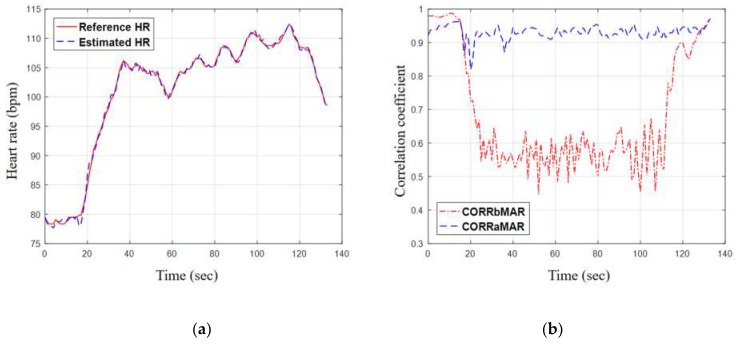
Taking subset 19 (the best case) for example to further analysis: (**a**) the contrast curves of HR estimation results and the reference HR values; (**b**) correlation curves against time.

**Figure 10:**
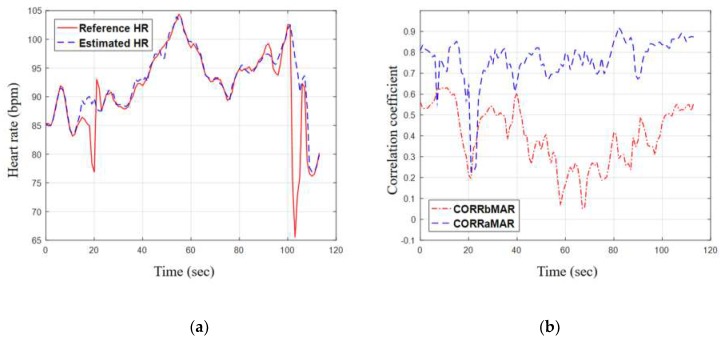
Taking subset 8 (the worst case) for example to further analysis: (**a**) the contrast curves of HR estimation results and the reference HR values; (**b**) correlation curves against time.

To illustrate the performance of the proposed method more clearly, the subset 19 (the best case) and the subset 8 (the worst case) were taken for examples to further analysis, as shown in Figure 9 and Figure 10. For the best case, Figure 9a shows the estimated HR stays close to the reference HR and is able to follow the instantaneous changes in reference HR. Figure 9b shows the two correlation values against time for subset 19. For the first 20 s where both hands/arms were motionless, the correlation values were both high and close to unity. After motion began (walking on the treadmill for subset 19), *CORR_bMAR_* dropped significantly to the range between 0.5 and 0.6. While *CORR_aMAR_* also dropped at the transition, it recovered quickly and always stayed around 0.9 during the intensive exercise. For the worst case, the estimated HR still stayed close to the reference most of the time, as shown in the Figure 10a. The wide differences at some time are caused by the sudden change of HR. Because of the excessive movement of right arm during the data acquisition, the left arm cannot be motionless completely, which results in the corrupted PPG reference signal.

## 4. Discussions and Conclusions

PPG sensors have been widely used in wearable health tracking, from the early HR monitoring to the present BP and SpO_2_ monitoring. However, the accuracy of PPG sensors during exercise is unsatisfactory due to the motion artifact. At present, many studies have proposed methods to improve the accuracy of HR monitoring, which ignore the morphological recovery of the PPG signal. It limits the application of PPG sensors. In this paper, a novel method is proposed for PPG MA removal during intensive exercise. On the one hand, an LMS-Newton-based cascaded ANC and unique HRF estimation scheme are applied to increase the accuracy of HR calculation. The mean AAE of HR during intensive exercise is improved from 11.47 bpm to 0.92 bpm, which verified preliminary on a widely-used open source data set of 12 subjects. On the other hand, the notch filters are employed here to recover the PPG components with the calculated HRF, which maintaining the morphological characteristics of PPG signals during intensive exercise. In the practical experiment, the proposed method achieves mean AAE result of 0.89 bpm, which shows the stability and strength of the proposed method for accurate HR calculation during intensive exercise. At the same time, the average correlation coefficient between recovered PPG signal and reference PPG signal reaching 0.86. The main features of PPG signal are restored, which is helpful to the future study of PPG sensors, such as wearable BP monitoring based on PPG. In addition, the proposed method has the advantage of real-time performance. It only costs an average of 0.83 ms to process the PPG signal with 25 Hz sampling rate. The proposed method provides a good basis for the improvement of the wearable PPG sensors.

## Figures and Tables

**Table 1:** Results of *HR_AAE_* with and without the cascaded adaptive noise cancellation (ANC) stage.

Subject	1	2	3	4	5	6	7	8	9	10	11	12	Mean
BPF only	8.80	26.12	17.27	6.23	1.28	4.61	2.06	2.95	0.36	40.99	13.49	13.44	11.47
BPF + ANC	4.18	1.51	1.24	0.89	0.67	1.22	2.20	0.49	0.34	4.68	0.85	0.81	1.59

**Table 2:** Results of *HR_AAE_* before and after applying the heart rate frequency tracking (HRFT) stage.

Subject	1	2	3	4	5	6	7	8	9	10	11	12	Mean
BPF +ANC	4.18	1.51	1.24	0.89	0.67	1.22	2.20	0.49	0.34	4.68	0.85	0.81	1.59
BPF +ANC+HRFT	1.46	1.17	1.12	0.89	0.67	1.22	0.90	0.49	0.34	2.24	0.85	0.81	1.01

**Table 3:** Results of *HR_AAE_* for the complete HRF estimation algorithm.

Subject	1	2	3	4	5	6	7	8	9	10	11	12	Mean
BPF +ANC+HRFT	1.46	1.17	1.12	0.89	0.67	1.22	0.90	0.49	0.34	2.24	0.85	0.81	1.01
Complete HR Estimation	1.09	0.87	1.20	0.81	0.67	1.15	0.73	0.49	0.34	2.06	0.87	0.78	0.92

**Table 4:** Comparison of the results of *HR_AAE_* for the open source data set.

**Subject**	**IEEE Access’16** [20]	**IEEE TMBE’16** [14]	**MUARD** [21]	**WFPV** [15]	**HSUM** [22]	**This Work**
1	1.16	1.70	1.17	1.25	0.76	1.09
2	1.07	0.84	0.93	1.41	0.92	0.87
3	0.80	0.56	0.70	0.71	0.95	1.20
4	1.13	1.15	0.82	0.97	1.19	0.81
5	0.98	0.77	0.88	0.75	0.70	0.67
6	1.29	1.06	0.97	0.92	0.61	1.15
7	0.88	0.63	0.67	0.65	0.87	0.73
8	0.81	0.53	0.74	0.97	0.59	0.49
9	0.55	0.52	0.49	0.55	0.53	0.34
10	3.18	2.56	2.69	2.06	0.75	2.06
11	0.79	1.05	0.81	1.03	1.50	0.87
12	0.72	0.91	0.77	0.99	2.47	0.78
Mean	1.11	1.02	0.97	1.02	0.99	0.92

**Table 5:** Detailed description for the motion patterns of the practical experiment.

**Subset**	**Number of Subjects**	**Motion Pattern**
1	1	Motionless (120 s)
2–10	9	Motionless (20 s) → Long-range Full-arm Swinging at 1 Hz (20 s) → Middle-range Full-arm Swinging at 1.5 Hz (20 s) → Long-range Full-arm Swinging at 1 Hz (20 s) → Middle-range Full-arm Swinging at 1.5 Hz (20 s) → Motionless (20 s)
11–16	6	Motionless (20 s) → Random Keyboard Hitting (20 s) → Left-right Wrist Swinging (20 s) → Left-right Forearm Swinging (20 s) → Up-down Forearm Swinging (20 s) →Motionless (20 s)
17–20	1	Motionless (20 s) → Walking on a treadmill at 3–6 km/h (80–100 s) →Motionless (20 s)

**Table 6:** Experimental results for the 20 data subsets of the self-built data set.

Subset	*HR_AAE_*	Average *CORR_bMAR_*	Average *CORR_aMAR_*
1	0.18	0.98	0.90
2	1.02	0.58	0.90
3	0.94	0.64	0.84
4	0.87	0.31	0.86
5	0.53	0.67	0.89
6	0.59	0.73	0.90
7	1.33	0.61	0.78
8	1.81	0.55	0.84
9	0.79	0.65	0.90
10	0.77	0.54	0.83
Mean(2–10)	0.96	0.59	0.86
11	0.49	0.58	0.89
12	1.12	0.54	0.80
13	1.29	0.44	0.75
14	1.05	0.34	0.74
15	1.41	0.60	0.80
16	0.53	0.77	0.92
Mean(11–16)	0.98	0.55	0.81
17	1.06	0.63	0.89
18	0.41	0.61	0.93
19	0.36	0.58	0.93
20	0.47	0.49	0.90
Mean(17–20)	0.57	0.58	0.91
Mean(2–20)	0.89	0.57	0.86
